# De novo disruptive heterozygous *MMP21* variants are potential predisposing genetic risk factors in Chinese Han heterotaxy children

**DOI:** 10.1186/s40246-022-00409-9

**Published:** 2022-09-19

**Authors:** Xi-ji Qin, Meng-meng Xu, Jia-jun Ye, Yi-wei Niu, Yu-rong Wu, Rang Xu, Fen Li, Qi-hua Fu, Sun Chen, Kun Sun, Yue-juan Xu

**Affiliations:** 1 Department of Pediatric Cardiology, Xinhua Hospital, Affiliated to Shanghai Jiao Tong University School of Medicine, Room 505, Scientific Building, Shanghai, 200092 China; 2 Department of Pediatrics, The First Affiliated Hospital of Zhengzhou University, Zhengzhou, 450052 China; 3 Scientific Research Center, Xinhua Hospital, Affiliated to Shanghai Jiao Tong University School of Medicine, Shanghai, 200092 China; 4 Department of Pediatric Cardiology, Shanghai Children’s Medical Center, Affiliated to Shanghai Jiao Tong University School of Medicine, Shanghai, 200127 China; 5 Medical Laboratory, Shanghai Children’s Medical Center, Affiliated to Shanghai Jiao Tong University School of Medicine, Shanghai, 200127 China

**Keywords:** Congenital heart disease, Heterotaxy syndrome, MMP21, Exome sequencing, Zebrafish

## Abstract

**Background:**

Heterotaxy syndrome (HTX) is caused by aberrant left–right patterning early in embryonic development, which results in abnormal positioning and morphology of the thoracic and abdominal organs. Currently, genetic testing discerns the underlying genetic cause in less than 20% of sporadic HTX cases, indicating that genetic pathogenesis remains poorly understood. In this study, we aim to garner a deeper understanding of the genetic factors of this disease by documenting the effect of different matrix metalloproteinase 21 (MMP21) variants on disease occurrence and pathogenesis.

**Methods:**

Eighty-one HTX patients with complex congenital heart defects and 89 healthy children were enrolled, and we investigated the pathogenetic variants related to patients with HTX by exome sequencing. Zebrafish splice-blocking Morpholino oligo-mediated transient suppression assays were performed to confirm the potential pathogenicity of missense variants found in these patients with HTX.

**Results:**

Three *MMP21* heterozygous non-synonymous variants (c.731G > A (p.G244E), c.829C > T (p.L277F), and c.1459A > G (p.K487E)) were identified in three unrelated Chinese Han patients with HTX and complex congenital heart defects. Sanger sequencing confirmed that all variants were de novo. Cell transfection assay showed that none of the variants affect mRNA and protein expression levels of *MMP21*. Knockdown expression of *mmp21* by splice-blocking Morpholino oligo in zebrafish embryos revealed a heart looping disorder, and mutant human *MMP21* mRNA (c.731G > A, c.1459A > G, heterozygous mRNA (wild-type&c.731G > A), as well as heterozygous mRNA (wild-type& c.1459A > G) could not effectively rescue the heart looping defects. A patient with the MMP21 p.G244E variant was identified with other potential HTX-causing missense mutations, whereas the patient with the MMP21 p.K487E variant had no genetic mutations in other causative genes related to HTX.

**Conclusion:**

Our study highlights the role of the disruptive heterozygous *MMP21* variant (p.K487E) in the etiology of HTX with complex cardiac malformations and expands the current mutation spectrum of *MMP21* in HTX.

**Supplementary Information:**

The online version contains supplementary material available at 10.1186/s40246-022-00409-9.

## Background

Early embryonic aberrant left–right asymmetric development leads to a broad spectrum of congenital malformations, including situs inversus and situs ambiguous (also called heterotaxy, HTX) [1, 2]. HTX is a serious congenital malformation characterized by abnormal positioning and morphology of the heart, lungs, and abdominal organs along the left–right axis, occurring in approximately 1/10000–1/5000 live births [3, 4]. More than 90% of HTX patients have complex congenital heart diseases (CHDs), including single ventricle (SV), single atrium (SA), transposition of the great arteries (TGA), double-outlet right ventricle (DORV), complete atrioventricular canal (CAVC), and total anomalous pulmonary venous connection (TAPVC), which account for approximately 3% of all CHDs [4, 5]. Patients with HTX and CHDs face significant complications, such as respiratory dysfunction, valve regurgitation, cardiac insufficiency, and challenging medical and surgical treatments [6–8]. Owing to its high clinical mortality and low postoperative survival rate, HTX has become the focus of many studies.

The pathogenesis of HTX is multifactorial and involves both genetic and environmental factors. The environmental factors include in utero retinoic acid exposure, type I maternal diabetes mellitus, and maternal cocaine use [9–11]. Although associations between these environmental factors and HTX have been documented, their exact roles remain poorly understood. On the other hand, high-throughput sequencing has identified many genetic factors that play vital roles in the pathogenesis of HTX, such as cilia proteins (DYX1C1, DNAH5, DNAH9, DNAH11, ZMYND10, C11ORF70, LRRC56, PKD2, NUP188, NEK2, FDZ3), NODAL and its relatives (NODAL, CITED2, ACVR2B, ZIC3, CFC1, GDF1, DAND5, LEFTYB, LEFTYA), and left–right organizer proteins (FGFR4, ZIC3, RAPGEF5, GALNT11) [12–22]. Hoverer, genetic testing scans out the underlying genetic cause in less than 20% of sporadic HTX cases, indicating that the genetic pathogenesis of HTX remains poorly understood.

MMP21 is a member of the matrix metalloproteinase (MMP) family and plays an important role in various physiological and pathological processes [23, 24]. The human *MMP21* gene is located on chromosome 10q26.2, contains 7 exons, and measures approximately 20 kb [25, 26]. *MMP21* regulates embryonic development, particularly neural development [26, 27]. Notably, emerging data have also confirmed its involvement in the pathogenesis of HTX. For example, ENU-induced mice *Mmp21* mutants showed laterality defects and complex CHDs [28, 29], while Morpholino oligo (MO)-mediated transient suppression or CRISPR/Cas9-mediated deletion of *mmp21* in zebrafish embryos caused cardiac looping defects [29, 30]. Therefore, exome sequencing will be useful for screening novel pathogenetic candidates, expanding the mutational spectrum of patients with syndromic, non-syndromic, and sporadic HTX, ultimately providing a better understanding of the contribution of susceptible alleles to disease penetrance and pathogenesis. HTX-related pathogenic homozygous and compound heterozygous variants of *MMP21* were detected in patients with HTX via exome sequencing [18, 19, 29–32]; however, no studies reported whether simple heterozygous MMP21 variants can cause HTX. Thus, scanning the *MMP21* variants in patients with HTX could help clarify the genotype–phenotype correlation and provide clues for genetic counseling.

In this study, we aim to garner a deeper understanding of the genetic factors of HTX by documenting the effect of *MMP21* variants on disease occurrence and pathogenesis. We used exome sequencing to detect potential pathogenic variants in a cohort of 81 patients with combined HTX and CHDs and 89 healthy controls. Three rare heterozygous non-synonymous *MMP21* variants (p.G244E, p.L277F, p.K487E) were documented in three unrelated patients with HTX and confirmed to be de novo mutations by Sanger sequencing. Two of these variants (p.G244E and p.K487E) were also proven to be deleterious in zebrafish MO rescue experiments. Our data expand the spectrum of *MMP21* variants associated with HTX and suggest that heterozygous *MMP21* variants may play a role in the pathogenesis of HTX and confer susceptibility to HTX with CHDs.

## Methods

### Ethical statement

These studies involving human participants were approved by the Medical Ethics Committee of Xinhua Hospital (NO. XHEC-C-2012–018) and Shanghai Children’s Medical Center (NO. SCMC-201015). Human peripheral blood samples were extracted voluntarily when donors signed the informed consent (or their parents/guardian if the donors were too young to consent). Zebrafish experiments were conducted under the approval of the Animal Ethics Committee of Xinhua Hospital, affiliated to Shanghai Jiao Tong University School of Medicine.

### Study population

From January 2012 to December 2016, we recruited 81 HTX patients with CHDs and 89 healthy children from Shanghai Children’s Medical Center and Xinhua Hospital. All patients were diagnosed using an echocardiogram, computed tomography, or magnetic resonance imaging. The patient cohort included 50 males and 31 females, with a mean age of 3.01 ± 2.60 years (range 12 days–15 years). All patients had cardiac defects including dextrocardia, mesocardia, left atrium isomerism, right atrium isomerism (RAI), conotruncal defects, and abnormal superior/inferior vena cava. Extracardiac malformations included bronchial inversus, polysplenia/asplenia, and dextrogastria (Table 1). The control cohort included 58 males and 31 females, with an average age of 4.75 ± 3.75 years (range 3 months–13 years). The controls were children presenting for routine health checkups. We informed the children and their parents about the background, purpose, and significance of our study. After obtaining the signed written informed consent of the children and their legal guardians, we included the children in the control group and extracted peripheral blood for exome sequencing. All study participants were Han Chinese and not related to one another. The study design conformed to the guidelines of the Declaration of Helsinki.

**Table 1:** Cardiac and extracardiac abnormalities of 81 HTX patients with CHDs in this study

Abnormalities	Number	Percentage (%)
*Sex*		
Male	50	61.73
Female	31	38.27
*Cardiac position*		
Levocardia	20	24.69
Dextrocardia	45	55.56
Mesocardia	16	19.75
*Atrial isomerism*		
Single atrium	22	27.16
Left atrial isomerism	3	3.70
Right atrial isomerism	27	33.33
*Ventricular isomerism*		
Single ventricle	28	34.57
*Bronchi*		
Bronchial inversus	16	19.75
*Spleen*		
Polysplenia	16	19.75
Asplenia	7	8.64
*Stomach*		
Dextrogastria	2	2.47
Abnormal superior/inferior vena cava	22	27.16

### Exome sequencing identification and Sanger sequencing validation

After obtaining informed consent, we strictly collected whole peripheral blood samples from all participants and stored them in EDTA tubes individually without sample mixup. The QIAamp DNA Blood Mini Kit (Qiagen, Hilden, Germany) was used to isolate genomic DNA. Exome sequencing was performed in 81 HTX patients and 89 control children. Specifically, exome sequencing was performed using a commercial provider (Shanghai Biotechnology Co., Ltd.). Briefly, the SureSelect Human All Exon V6 kit (Agilent Technologies, Santa Clara, CA, USA) and Illumina HiSeq 2500 platform (Shanghai Biotechnology Co., Ltd., Shanghai, China) were used, and raw sequencing reads were compared to the reference human genome (hg19). The mapping ratio of the 170 samples included in this study (case and control) was above 99%, and the average sequencing depth was > 25X. Among all bases in the captured target area, the percentage of coverage ≥ 10 was 98%–99.99%. Exome sequencing data were filtered based on the following criteria: (1) the mutation genotype included exonic non-synonymous, coding indels, splice-site variants, or frameshift; (2) the average depth of sequencing was > 10X; (3) minor allele frequency was < 1% or not found in population-based databases including SNP database at NCBI (http://www.ncbi.nlm.nih.gov/), 1000 Genomes (http://www.1000genomes.org/), ESP6500 (http://evs.gs.washington.edu/EVS/), ExAC (http://exac.broadinstitute.org/), and gnomAD (http://gnomad.broadinstitute.org/).

Sanger sequencing was performed for validating the candidate variants of *MMP21* and confirming that all variants were de novo. The PCR primers were designed to amplify the coding regions, including the candidate variants, using Primer premier5 software (Additional file 1: Table S1). We added 250 ng of each genomic DNA sample and 1 μM of each primer to 20 μl of 1 × MyTaq™Mix (Bioline USA Inc.). The PCR conditions were as follows: 98 °C for 2 min; 30 cycles at 98 °C for 20 s, Tm for 30 s, 72 °C for 45 s, and 72 °C for 10 min. We used 1% agarose gel staining with GelRed (Biotium, USA) to analyze 2 μl of PCR products. The residual PCR products were sequenced using an ABI 3730XL sequencer (Applied Biosystems, USA), and the results were compared to reference *MMP21* cDNA sequences from NCBI (#NM_147191.1) using the GenBank BLAST program (http://blast.ncbi.nlm.nih.gov/Blast.cgi).

### In silico analysis

Different in silico pathogenicity prediction tools include MutationTaster (http://www.mutationtaster.org/), PROVEAN (http://provean.jcvi.org/), and Polyphen2 (http://genetics.bwh.harvard.edu/pph2/) were used to evaluate possible pathogenic effects of the identified variants (Table 2). Three-dimensional structural models of wild-type (WT) and mutant MMP21 proteins were constructed using SWISS-MODEL (https://www.swissmodel.expasy.org), and the effects of mutant protein configuration changes were analyzed using HOPE (http://www.cmbi.ru.nl/hope/).

**Table 2:** Clinical information and MMP21 variant characteristics in HTX patients with complex cardiac malformations

Patient ID	Gender	Age (year)	Diagnosis of HTX	Diagnosis of cardiovascular malformation	Base change	Amino acid change	SIFT	Mutation taster	PolyPhe2 _HDIV	1000 Genomes allele frequency	gnomAD allele frequency
P58	F	3	Dextrocardia	TGA/VSD/PS	c.G731A	p. G244E	0.007 (D)	1.0 (DC)	0.995 (D)	–	–
P7	F	1	Dextrocardia/RAI/CSS	PLSVC/SA/SV/TAPVC/PS/RAA	c.C829T	p. L277F	0.003 (D)	1.0 (DC)	0.997 (D)	–	–
P61	M	10	RAI/CSS	PAD/AVSD/SV	c.A1459G	p. K487E	0.011 (D)	0.01 (N)	0.849 (P)	–	0.00005915

*F* female; *M* male; *RAI* right atrial isomerism; *CSS* cardiosplenic syndrome; *TGA* transposition of great arteries; *VSD* ventricular septal defect; *PS* pulmonary stenosis; *PLSVC* persistent left superior vena cava; *SA* single atrium; *SV* single ventricle; *TAPVC* total anomalous pulmonary venous connection; *RAA* right aortic arch; *PAD* pulmonary artery dysplasia; *AVSD* atrioventricular septal defect; *D* damaging; *DC* disease causing; *N* polymorphism; *P* possibly damaging

### Alignment of multiple MMP21 protein sequences

The MMP21 amino acid sequences of various species, including Homo sapiens (human), Xenopus tropicalis (frog), Canis lupus familiaris (dog), Danio rerio (zebrafish), Mus musculus (house mouse), Macaca mulatta (rhesus monkey), Rattus norvegicus (rat), and Pan troglodytes (chimpanzee) were downloaded from the UniProt database (http://www.uniprot.org). The Clustal X software (http://www.clustal.org) was used for sequence alignment.

### Plasmids and mutagenesis

The pCMV3-Human *MMP21* expression vector and pGEM-T-Human *MMP21* clone vector containing human *MMP21* cDNA (NCBI RefSeq NM_147191.1) were purchased from Sino Biological (Sino Biological, China). The variants p.G244E (NM_147191.1: c.G731A), p.L277F (NM_147191.1: c.C829T), and p.K487E (NM_147191.1: c.A1459G) were introduced into the WT pCMV3-Human *MMP21* vector and pGEM-T-Human *MMP21* vector using site-mutagenesis primers (Additional file 1: Table S1) via PCR. The accuracy and integrity of all plasmids were verified by Sanger sequencing.

### Cell culture and transfection

Human embryonic kidney 293 T (HEK-293 T) cells obtained from the Type Culture Collection of the Chinese Academy of Sciences (Shanghai, China) were cultured in Dulbecco’s modified Eagle’s medium (HyClone, USA) supplemented with 1% penicillin–streptomycin (Gibco, USA) and 10% fetal bovine serum (MP Biomedicals, USA) under a humidified atmosphere (95% air and 5% carbon dioxide) at 37 °C. When cell density in the 6/12-well plate reached 80%, plasmids were transfected into HEK-293 T cells using FuGene HD (Promega, USA) according to the manufacturer’s protocol. Homozygous and heterozygous variants of MMP21 were simulated using the following two transfection schemes: (1) 1 µg of blank, WT, or mutant MMP21 vectors were separately transfected into HEK-293 T cells; (2) 1 µg of blank, WT, or WT and mutant MMP21 vectors (1:1) were transfected into HEK-293 T cells.

### Quantitative real-time polymerase chain reaction

Total RNA was extracted from HEK-293 T cells using TRIzol reagent (Invitrogen, USA) after transfection for 36 h. Zebrafish embryos were crushed using a magnetic bead homogenate pulverizer and total RNA was extracted using TRIzol reagent after microinjection of control MO and splice-blocking Morpholino oligo (SB-MO) for 30 h. Subsequently, PrimeScript RT Master Mix (TaKaRa, Japan) was used for reverse transcription. Quantitative real-time amplification was performed using SYBR Premix Ex Taq (TaKaRa, Japan) on an Applied Biosystems 7500 system. Primers were synthesized by Shanghai Sangon Biotechnology Co., Ltd. (Additional file 1: Table S1). The 2^−ΔΔCt^ method was used to calculate the relative expression of MMP21, and human 18sRNA and zebrafish actin were used as internal references [33].

### Western blot

After transfection for 48 h, proteins from HEK-293 T cells were extracted using RIPA lysis buffer (Beyotime, China) supplemented with PMSF (1:100). Proteins were then separated by sodium dodecyl sulfate–polyacrylamide gel electrophoresis (SDS-PAGE) and transferred electrophoretically onto polyvinylidene fluoride membranes (Millipore, USA). The membranes were subsequently incubated with 5% skim milk for 1.5 h at room temperature and then with anti-MMP21 (1:650, 55,289-1-AP, Proteintech) and anti–actin antibodies (1:2000, AF5001, Beyotime) overnight at 4 °C. Next, the membranes were incubated with horseradish peroxidase-labeled goat anti-rabbit secondary antibody (1:5000) and goat anti-mouse secondary antibody (1:5000) for 1.5 h at room temperature and detected with Immobilon Western Chemiluminescent HRP Substrate (Millipore, USA) using a chemiluminescence system (BioRad).

### Transcriptional synthesis of mRNA in vitro

The WT and mutant pGEM-T-human *MMP21* vectors were used as templates. NotI restriction enzymes were selected based on the location of the T7 promoter in the plasmid map to perform single enzyme digestion of the vector and linearize the plasmid. We generated WT and mutant human *MMP21* mRNAs by in vitro transcription, mRNA capping, poly-A tail addition, and mRNA purification, using the mMESSAGE mMACHINE™ T7 Transcription Kit (Thermo Fisher Scientific, USA) according to the manufacturer’s protocol. Human *MMP21* mRNA was verified using 1% agarose gel electrophoresis.

### Zebrafish maintenance

WT AB strains of zebrafish were obtained from China Zebrafish Resource Center and bred under standard laboratory conditions as previously reported [34]. The water in the feeding system was purified circularly using water filtration equipment and maintained at 28 ± 1 °C with a 14 h/10 h light/dark cycle.

### Morpholinos and microinjection in zebrafish

SB-MO was designed and synthesized by Gene Tools (OR, USA) to suppress *mmp21* (NM_001317753.1) splicing and translation by targeting the exon 3–intron 3 junction (sequences: 5ʹ-AAATGTGCGATTTAAAACCTGTGCA-3ʹ). MO (5ʹ-CCTCTTACCTCAGTTACAATTTATA-3ʹ) was used as a negative control. Sexually mature male and female zebrafish were separately placed in the same mating box at a 1:1 ratio in the evening and mated the following morning. Zebrafish eggs were collected and arranged in the microinjection mold groove and a working solution was injected into the yolk sac of embryos using a pressure microinjection apparatus (Warner PLI-100A, USA) when the embryos were at the 1–2 cell stage. SB-MO efficiency was verified by RT-qPCR of *mmp21* cDNA (NM_001317753.1) obtained from whole embryos 30 h after microinjection. A preliminary experiment was then conducted to determine the optimal interventional concentration of SB-MO. Human *MMP21* mRNA (300 ng/μL) was used to rescue the phenotype of SB-MO. Since variants found in the patients were heterozygous, we mixed WT and mutant *MMP21* mRNAs (1:1) for microinjection to achieve a heterozygous state. Each microinjection had a volume of approximately 2–5 nL, and 470–570 embryos were injected in each group. Embryos were then regularly observed, and their phenotypes within each group were recorded at 48 h post-fertilization (hpf). The phenotypes recorded include death, cardiac location, looping abnormality, pericardial edema, tail deformity, and spinal curvature. Zebrafish embryos were imaged using an SMZ25 microscope (Nikon, Tokyo, Japan) equipped with a digital camera.

### Whole-mount in situ hybridization (WISH)

Primers (Additional file 1: Table S1) were designed to amplify zebrafish *cmlc2* (NM_131329.3) by PCR via 2 × TransTaq^®^ High Fidelity (HiFi) PCR SuperMix II (TransGen Inc., China). The pGEM^®^-T Easy Vector System (Promega, USA) was used to construct the *cmlc2* antisense probe plasmid. NcoI restriction enzyme was selected to perform single-enzyme digestion of the vector and linearize the plasmid. Digoxigenin (DIG)-labeled *cmlc2* antisense RNA probes were transcriptionally synthesized in vitro using an SP6 RNA Polymerase (Promega, USA). The 48 hpf embryos preserved by methanol dehydration were gradually rehydrated, fixed with 4% PFA, and digested with proteinase K (10 μg/mL). After pre-incubation with hybridization buffer at 65 °C for 4 h, the embryos were incubated with Dig-labeled *cmlc2* RNA antisense probes at 65 °C overnight. Next, after gradient washing, the 48 hpf embryos were immersed in anti-DIG antibody (Roche, Germany) and shaken slowly at room temperature for 1 h and then at 4 °C overnight. Finally, immunopositive alkaline phosphatase signals of the 48 hpf embryos were visualized using BM Purple (Sigma, Japan) after washing. An SMZ25 microscope (Nikon, Japan) equipped with a digital camera was used to capture images.

### Statistical analysis

All measurement data are presented as the mean ± standard error (SEM). The experiments were independently repeated at least three times. Statistical analyses were performed using GraphPad Prism 8. The results of RT-qPCR and western blotting were analyzed using one-way ANOVA with Tukey’s multiple comparison test. The percentage of zebrafish embryonic phenotypes was compared between groups using the chi-square test in zebrafish MO rescue experiments (and Fisher’s exact test). Statistical significance was set at *p* < 0.05.

## Results

### Three de novo heterozygous *MMP21* variants were identified in three unrelated HTX patients with CHDs

Using exome sequencing, we reached a molecular diagnosis for 25 of the 81 probands (30.9%). Briefly, we identified a total of 28 missense variants pathogenic (or likely pathogenic) in 11 genes known to be associated with HTX (*DNAH5*, *DNAI1*, *GALNT11*, *MEGF8*, *MMP21*, *NODAL*, *NPHP4*, *PKD1L1*, *ROCK2*, *SHROOM3*, *SMAD2*) (Additional file 3: Table S3). Additionally, three rare heterozygous non-synonymous *MMP21* variants were identified in three unrelated Chinese Han patients with HTX and complex cardiac malformations: p.G244E in dextrocardia, p.L277F in dextrocardia and RAI, and p.K487E in RAI (Table 2; Additional file 2: Table S2). The proportion of patients with *MMP21* variants in the clinical cohort was 3.7% (3/81). All *MMP21* variants were absent in the 89 controls and the related parents in our study.

**Fig. 1:**
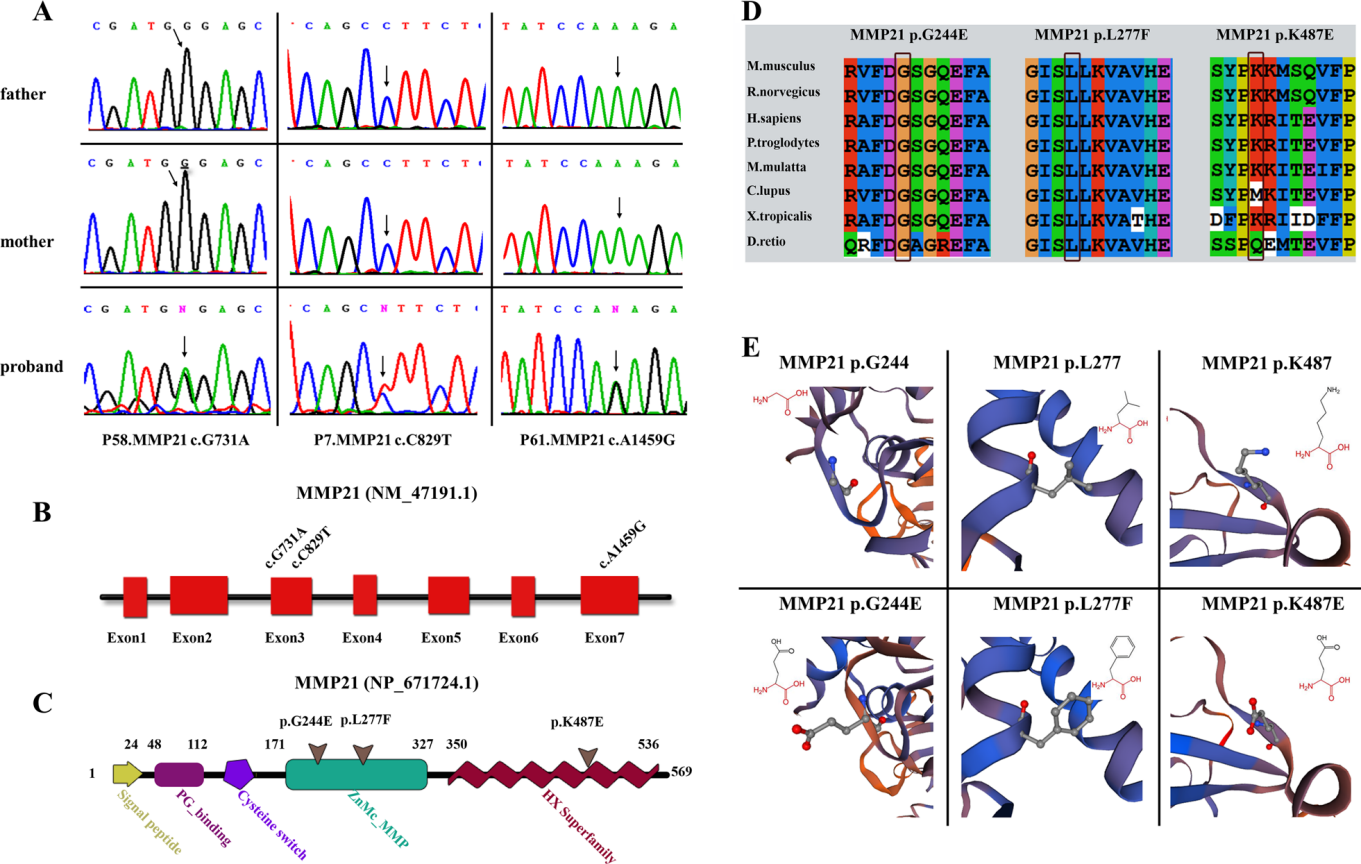
Validation and in silico analysis of *MMP21* variants found in HTX patients with complex CHDs. **A** Sanger sequencing chromatograms of three heterozygous missense *MMP21* variants in patients with HTX and complex CHDs and the corresponding normal sequences in related parents. Arrows represent heterozygous nucleotide changes. **B, C** Schematic diagrams of *MMP21* gene and protein showing the location of the identified *MMP21* variants. The base substitutions are in exon3 and exon7, respectively (**B**). The amino acids consist of the domains of the ZnMC_MMP and HX superfamily (**C**). **D** Clustal X alignment of amino acid mutations in MMP21 among different species indicates that all mutations are highly conserved in vertebrates. **E** Schematic diagram of the three-dimensional structure and corresponding amino acid residues at the mutation sites of WT (left panel) and mutant (right panel) MMP21 proteins that were reconstructed by SWISS-MODEL. Amino acid substitutions in the variants p.G244E and p.K487E resulted in changes in acidity or basicity, which may affect the function of the mutant domain. The amino acid residue in the original position of the variant p.L277F was replaced by a residue that occupies a larger space volume, which may affect the stability of the protein spatial structure

Among the three rare heterozygous missense *MMP21* variants, p.K487E (NM_147191.1:c.1459A > G, rs779248373) was found in the NCBI SNP, ExAC, and gnomAD databases, and its MAF was 0.0009563 in East Asians, p.L277F (NM_147191.1:c.829C > T, rs1850462241) was only found in the NCBI SNP database and its MAF was 0.000011 in TOPMed and notably, variant p.G244E (NM_147191.1:c.731G > A) has never been previously reported. Sanger sequencing chromatograms of the identified *MMP21* variants in the probands and their parents are shown in Fig. 1A.

Several potential HTX causative variants were also screened in patients harboring *MMP21* mutations (Additional file 2: Table S2). In the MMP21-G244E patient, we identified two additional potential HTX-causing missense mutations in Rho-associated coiled-coil containing protein kinase 2 (ROCK2, NM_004850, c.345G > C, p.Q115H, rs564194657) and nucleoporin 188 (NUP188, NM_015354, c.5018C > T, p.P1673L, rs568477773).

### The mutants might affect the function of MMP21 by in silico analysis

The three *MMP21* variants are distributed in exon3 and exon7 (Fig. 1B), and the corresponding amino acid substitutions are in two conserved domains of the zinc-dependent MMP catalytic domain (ZnMC_MMP) and heme-like repeat sequence (HX superfamily) (Fig. 1C). High pathogenicity scores of the three rare mutation loci, calculated using multiple online bioinformatics software (Mutation taster, PROVEAN, Polyphen2), highlighted the deleterious effect of all mutations (Table 2; Additional file 2: Table S2). The amino acid substitution site was highly conserved in multiple vertebrates, as shown in the amino acid sequence alignment of MMP21 protein using Clustal X software (Fig. 1D), indicating that amino acid substitutions might result in functional alterations of MMP21 protein.

To further investigate the functional effect of the variants, we used SWISS-MODEL to construct the three-dimensional structure of the WT and mutant MMP21 proteins (Fig. 1E). Amino acid substitutions of the p.G244E and p.K487E variants changed the acidity and basicity of the amino acids, which may affect spatial configuration and alter protein function. Phenylalanine (F), which contains a benzene ring, replaced the branched leucine (L) in the p.L227F variant potentially affecting the stability of the catalytic domain. Further analysis of the three-dimensional configuration of the mutant protein by HOPE revealed that the original amino acid residues were replaced by residues with larger spatial volumes (Fig. 1E), which may affect the stability of the protein spatial structure. The physicochemical properties of the mutant amino acid may not be suitable for its spatial position, which affects its interaction with other parts of the protein’s three-dimensional structure (Fig. 1E).

### The expression of MMP21 was not affected by the mutations

**Fig. 2:**
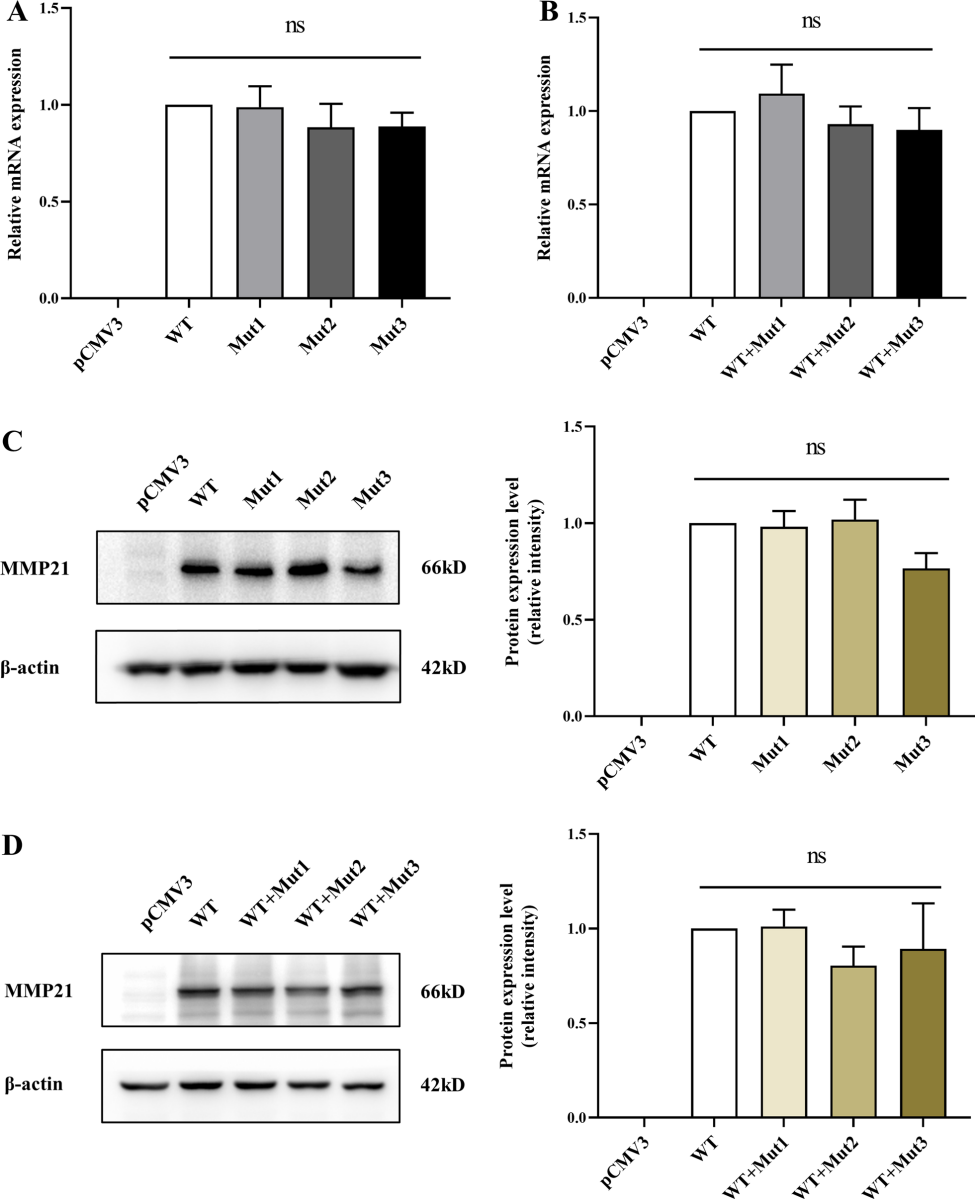
The variants do not disrupt the expression of *MMP21*. The blank (pCMV3), *MMP21* WT, or mutant vectors (Mut1, c.G731A; Mut2, c.C829T; Mut3, c.A1459G) were separately transfected or co-transfected (WT/mutant = 1:1) into HEK-293 T cells. **A, B** Relative mRNA expression of *MMP21* in HEK-293 T cells transfected with plasmids for 36 h was assessed by RT-qPCR. 18sRNA was used as an internal control. The RNA levels in the WT group were set at 1. There was no significant difference in *MMP21* mRNA expression between the WT and mutant groups (all data are shown as the mean ± SEM, and one-way ANOVA with Tukey’s multiple comparisons test was performed for statistical calculation of *MMP21* mRNA expression between the WT and mutant groups, *n* = 3 independent experiments). **C, D** Relative MMP21 protein expression in HEK-293 T cells transfected with plasmids for 48 h was evaluated using western blot analysis. β-Actin was used as an internal control (left panel). The intensity quantitation of MMP21 WT and mutant protein expression levels were normalized to β-actin expression using ImageJ software (right panel). Quantitative results revealed no significant difference in MMP21 protein expression between WT and mutant groups (all data are shown as the mean ± SEM; one-way ANOVA with Tukey’s multiple comparisons test was used for statistical calculation of MMP21 protein expression between WT and mutant groups, *n* = 3 independent experiments)

To explore the effect of the identified variants on *MMP21* expression, we constructed mutant *MMP21* expression vectors (pCMV3-*MMP21*-mut1 (p.G244E), pCMV3-*MMP21*-mut2 (p.L277F), and pCMV3-*MMP21*-mut3 (p.K487E)). Blank (pCMV3), *MMP21* WT, and mutant vectors were separately transfected or co-transfected into HEK293 cells. RT-qPCR results indicated that neither heterozygous nor homozygous mutations affect the transcription level of *MMP21* (Fig. 2A, B). Quantitative results of the western blot analysis showed no statistically significant change in dosage between WT and variant MMP21 proteins in both homozygous and heterozygous states (Fig. 2C, D); however, MMP21 protein expression level in the separately transfected Mut3 group showed a downward trend (*p* = 0.0529).

### Mutant *MMP21* mRNA c.731G > A (mut1) and c.1459A > G (mut3) failed to rescue the zebrafish heart looping disorder induced by mmp21 knockdown via SB-MO.

**Fig. 3:**
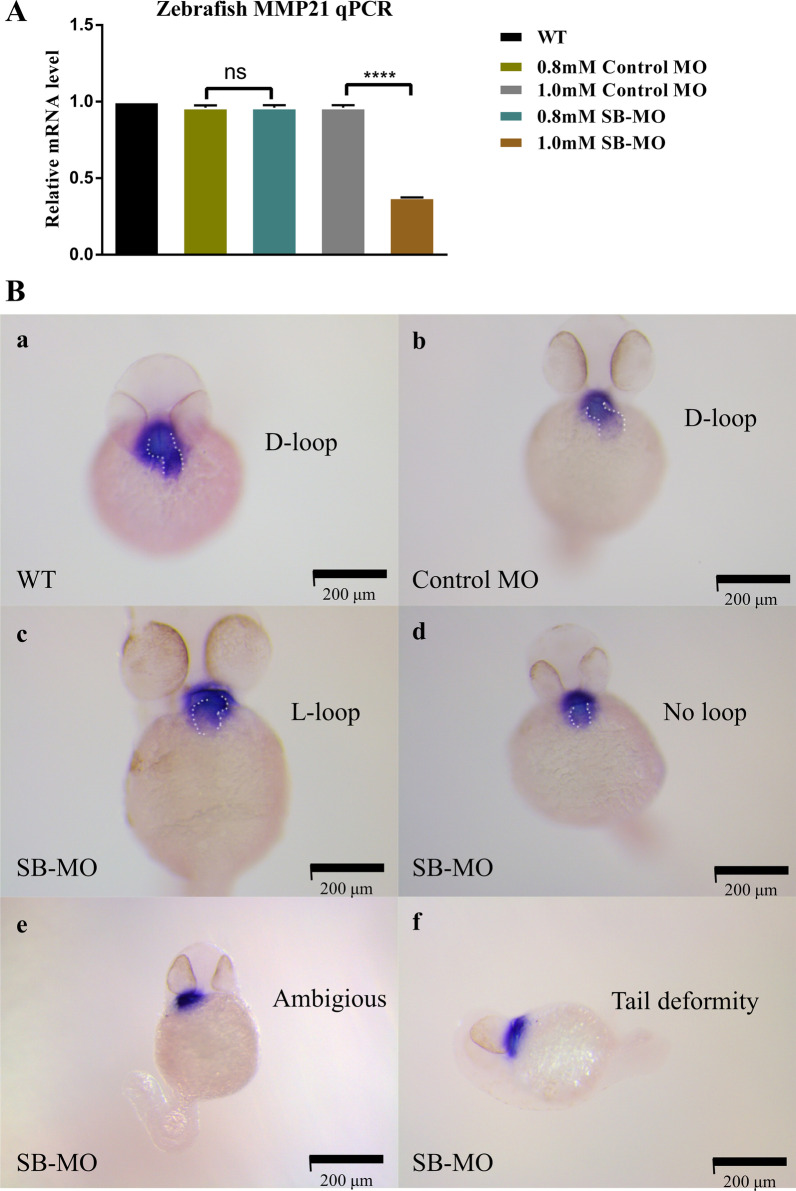
*mmp21* knockdown induced by SB-MO caused heart looping disorder in zebrafish embryos. **A** Relative *mmp21* mRNA expression in zebrafish embryos injected with SB-MO and standard control MO at concentrations of 0.8 mM and 1.0 mM for 30 h was assessed by RT-qPCR. RT-qPCR results showed that microinjection of SB-MO (1.0 mM) significantly decreased the *mmp21* mRNA expression in zebrafish embryos compared to the control MO group (data are shown as the mean ± SEM, one-way ANOVA with Tukey’s multiple comparisons test was used for statistical calculation, *n* = 3 independent experiments, ns: nonsignificant, ****p < 0.0001). **B** Whole-mount in situ hybridization using a cmlc2 antisense probe to detect cyclization of 48 hfp embryonic cardiac tubes after MO injection. **a, b** The WT and control MO groups showed normal heart looping (D-loop). **c, d, e** Zebrafish embryos in the SB-MO group formed an abnormal L-loop on the left side or manifested as ambiguous looping or no loop. **f** Some zebrafish embryos in the SB-MO group showed tail deformity, which may be caused by side effects of SB-MO. Scale bar, 200 μm

Engineered mutants or transient suppression using Morpholinos for target genes in zebrafish have broadened our understanding of the pathogenic mechanisms of HTX. To investigate whether the identified mutations affected MMP21 protein function and their role in left–right axial development, an *mmp21* knockdown zebrafish model was constructed by SB-MO microinjection. We carried out a preliminary experiment to investigate the proper concentration of the standard control MO and SB-MO for microinjection. RT-qPCR suggested that it was 1.0 mM, and showed that SB-MO could effectively block the splicing of *mmp21* in zebrafish (Fig. 3A). Also, most zebrafish embryos died in both the experimental and control groups when the working solution concentration reached 1.5 mM, suggesting that MO side effects positively correlated with an increase in dosage.

Zebrafish *cmlc2* encodes cardiac myosin light chain 2, which is an indispensable component for the assembly of thick myofilaments and acts as a heart-specific protein marker [35]. The antisense RNA of *cmlc2* was used as a probe to accurately display the outline and cyclization direction of the zebrafish heart tube. Consistent with previous studies [29, 30], in situ hybridization analysis at 48 hfp indicated that the heart looping direction in WT and MO groups was on the right side (D-loop), whereas the SB-MO group showed abnormal heart looping (such as L-loop or no looping) and contour deformity (Fig. 3B). In addition, some embryos exhibited tail deformities and spinal curvatures in the SB-MO group (Fig. 3B).

**Fig. 4:**
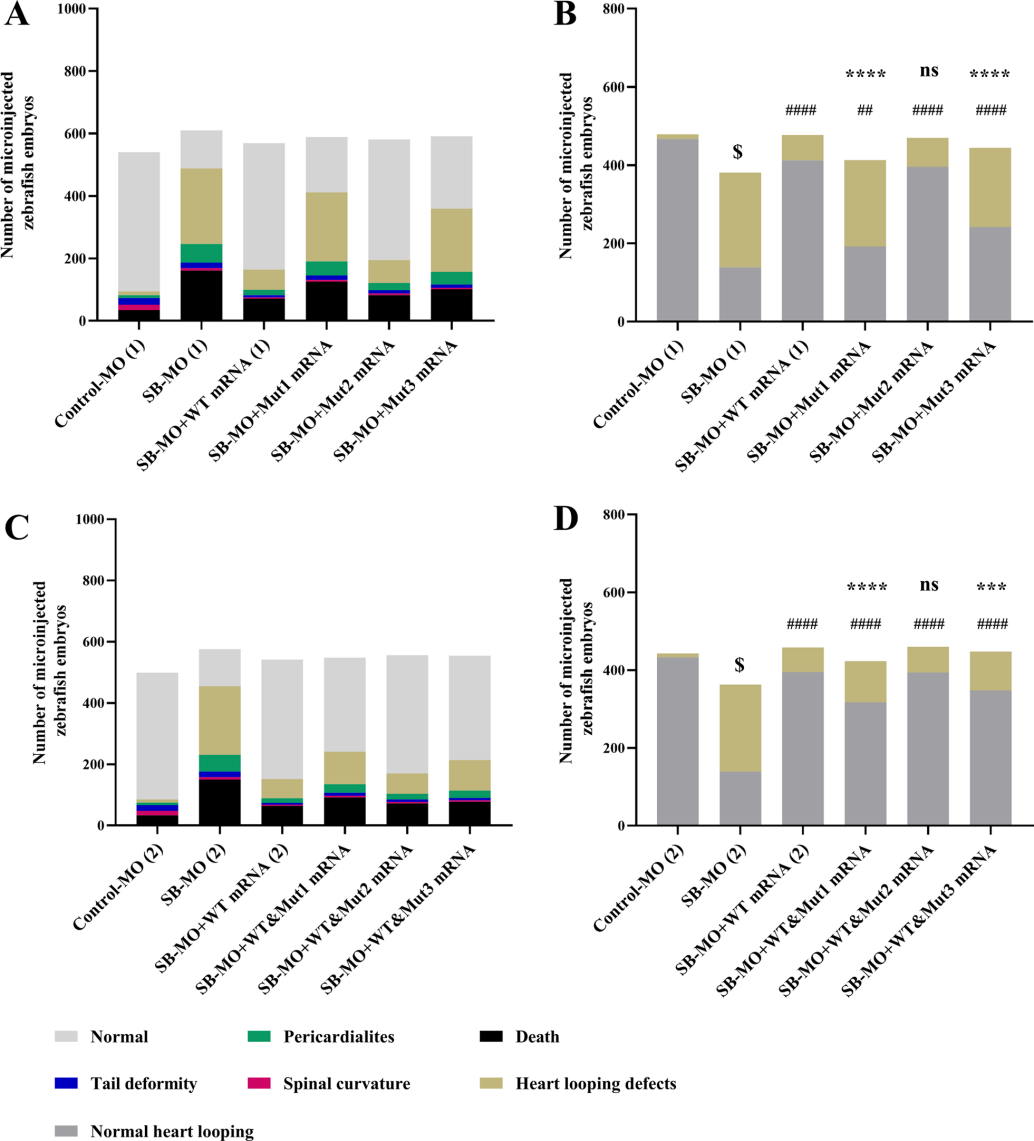
Microinjection of *MMP21* mRNA c.731G > A and c.1459A > G failed to rescue the zebrafish heart looping disorder. The WT and mutant human *MMP21* mRNAs synthesized using in vitro transcription were separately microinjected or co-microinjected (WT/mutant = 1:1) into 1–2 cell stage zebrafish embryos accompanied by SB-MO to compensate for the *mmp21* knockdown. The zebrafish heart looping disorder phenotype at 48 hfp was observed and analyzed. Heart tube looping abnormalities include L-loop, ambiguous looping, or no looping. Left panels show all phenotypes after separate microinjection or co-microinjection of WT and mutant *MMP21* mRNAs to zebrafish embryos. The right panels show normal and abnormal heart looping phenotypes. **A, B** Compared to the SB-MO group, *MMP21* WT mRNA effectively reduced the number of malformed phenotypes. Mut1 and Mut3 could not effectively compensate for the effects of *mmp21* knockdown. The effect of Mut2 was similar to that of the WT mRNA. **C, D** When co-microinjected with the WT and mutant mRNAs at a 1:1 ratio, no statistical difference in the malformation rate between the WT&Mut2 group and the WT mRNA group was noted. The malformation rate of the WT&Mu1 and WT&Mu3 groups was significantly reduced compared to that of the SB-MO group; however, it remained significantly higher than that of the WT mRNA group (chi-square test was used for statistical calculation, $ *p* < 0.0001 versus control MO group, #### *p* < 0.0001 versus SB-MO group, ## *p* < 0.01 versus SB-MO group, **** *p* < 0.0001 versus WT mRNA group, *** *p* < 0.001 versus WT mRNA group, ns: nonsignificant)

Next, we investigated whether *MMP21* variants could rescue abnormal zebrafish heart looping induced by *mmp21* knockdown via SB-MO. To do so, WT and mutant pGEM-T-human *MMP21* vectors were used as templates to synthesize mRNA using in vitro transcription. WT and mutant *MMP21* mRNA at a concentration of 300 ng/µL was separately microinjected into 1–2 cell stage zebrafish embryos accompanied by SB-MO to compensate for *mmp21* knockdown. In the early stage of zebrafish cardiac development, the vital point of the heart tube begins to bend toward the right side at 36 hpf [36]. The heart looping of zebrafish embryos was unambiguously observed under a light microscope at 48 hpf (Fig. 4A). The incidence of heart looping defects was 2.51% in the control MO group and 63.52% in the SB-MO group. Compared with the SB-MO group without human *MMP21* mRNA complement, *MMP21* WT mRNA effectively reduced the incidence of malformed phenotypes (13.63% vs. 63.52%, *p* < 0.0001). However, a statistically significant difference compared to the control MO group (13.63% vs. 2.51%, *p* < 0.0001) remained. Interestingly, Mut1 (c.731G > A: p.G244E) and Mut3 (c.1459A > G: p.K487E) could not as effectively compensate for the effect of *mmp21* blockade as the *MMP21* WT mRNA (54.96% vs. 13.63%, p < 0.0001; 52.25% vs. 13.63%, *p* < 0.0001, respectively) (Fig. 4A). On the other hand, Mut2 (c.829C > T: p.L277F) rescued the phenotypes caused by *mmp21* SB-MO, similar to *MMP21* WT mRNA (15.74% vs. 13.63%, p = 0.3572) (Fig. 4A).

### WT&Mut1(c.731G > A) and WT&Mut3(c.1459A > G) heterozygous *MMP21* mRNA could not fully rescue the abnormal left–right pattern caused by *mmp21* knockdown in zebrafish.

Since all three *MMP21* mutations detected in patients were heterozygous, we wondered whether heterozygosity affected WT MMP21 protein function. WT and mutant human *MMP21* mRNA mix (WT/mutant = 1:1) was co-microinjected into *mmp21* MO-knockdown zebrafish. The rescue experiment showed that WT&Mut2 (c.829C > T) heterozygous *MMP21* mRNA mix could rescue the phenotypes caused by *mmp21* knockdown similar to homozygous WT *MMP21* mRNA (14.36% vs. 13.76%, *p* = 0.7962). The WT&Mut1 (c.731G > A) and WT&Mut3 (c.1459A > G) heterozygous *MMP21* mRNA mix could partially rescue the phenotypes, compared to the homozygous Mut1 and Mut3 *MMP21* mRNA; however, the malformation rate remained significantly higher than that of homozygous WT *MMP21* mRNA (WT&Mut1: 25.06% vs. WT: 13.76%, *p* < 0.0001; WT&Mut3: 22.32% vs. WT: 13.76%, *p* = 0.0008) (Fig. 4). These results suggested that MMP21-G244E and MMP21-K487E may have a dominant-negative effect and increase susceptibility to abnormal left–right patterning, supporting their functional and clinical relevance.

## Discussion

MMPs are a family of zinc-dependent endopeptidases critical for maintaining tissue allostasis under normal physiological conditions. ProMMPs are cleaved into their active forms and subsequently degrade various protein components of the extracellular matrix (ECM) [37]. Thus, MMPs are multifunctional proteins involved in tissue remodeling during various physiological processes (such as embryogenesis, morphogenesis, and angiogenesis) as well as pathological conditions (such as cancer, myocardial infarction, and osteoarthritis) [38, 39]. Twenty-eight members of the MMP family have been reported in vertebrates, whereas at least 23 have been detected in humans [38]. MMPs are further divided into gelatinases, matrilysin, collagenases, stromelysins, membrane-type MMPs, and other MMPs, depending on their substrate and structural domains [40].

Studies have shown that MMP1 and MMP2 are involved in modulating membrane motility and lumenogenesis during heart development in Drosophila flies [41]. MMP21, on the other hand, is a multifunctional secreted furin-containing MMP. Exploring its role in embryonic left–right axis establishment and cardiogenesis could potentially expand the functional profile of this protein family. Previous animal- and human-based studies have demonstrated that dysfunctional MMP21 protein predisposes patients to HTX [18, 19, 29–32]. Trio family pedigrees analysis using exome sequencing facilitates the identification of genetic alterations such as single nucleotide variants, copy number variations, and insertions/deletions (indels), which further validates the causal mutations in inherited diseases. In this study, three de novo heterozygous *MMP21* variants (p.G244E, p.L277F, and p.K487E) were detected in three unrelated patients with HTX and complex CHDs by exome sequencing and Sanger sequencing. None of these variants affected MMP21 expression. Two of them (MMP21-G244E and MMP21-K487E) were loss-of-function mutations in both homozygous and heterozygous states based on zebrafish rescue experiments. Our results suggest that these two variants confer susceptibility to abnormal left–right patterning as well as abnormal cardiac development and may be involved in the pathogenesis of HTX. Functionally compromised MMP21 expression may underlie the occurrence of HTX and various congenital cardiac malformations in humans.

We also synthesized mutant *MMP21* mRNA in vitro to rescue morphants. Zebrafish rescue experiments showed that heterozygous WT and p.G244E MMP21 mRNA mix as well as WT and p.k487E MMP21 mRNA mix could partially rescue the phenotypes caused by mmp21 knockdown via SB-MO. These results suggest that normal functioning MMP21 might play an important role in establishing the left–right axis and heart looping during early embryonic development in a quantity- and quality-dependent manner.

Previous studies have confirmed that MMPs are regulated at multiple levels, including mRNA transcription, conversion of the proenzyme to the active form, and inhibition of endogenous tissue inhibitors of metalloproteinases (TIMPs). Initially, MMPs are synthesized as pre-pro-MMPs and are then transformed into inactive pro-MMP by cleaving the signal peptide during translation. Our data indicated that the variants did not affect the transcription and translation of MMP21, suggesting that they may lead to the pathologic phenotype by affecting MMP21 activity. MMP21 consists of a pro-peptide, furin-like motif, catalytic metalloproteinase domain, hinge region, and hemopexin domain in the C-terminal [22]. The furin-like pro-protein convertase recognition sequence is the basis for intracellular activation of MMP21 through furin [42]. The identified variants are located in the catalytic metalloproteinase and hemopexin domains and thus may not affect the furin activation process. Tissue inhibitors of metalloproteinases (TIMPs) are endogenous protease inhibitors that regulate MMPs activation and function by binding to them in a 1:1 stoichiometric ratio [25] decreasing their ability to degrade ECM proteins. The catalytic metalloproteinase domain contains a zinc finger module (HEXXHXXGXXH), which forms the basis of enzymatic hydrolysis of MMPs. TIMP wedges into the catalytically active site cleft of MMPs and inhibits their activity and/or activation [25]. The MMP21-G244E and MMP21-L277F variants were mapped to highly the conserved catalytic domains (Fig. 1D). The MMP21-G244E variant in the catalytic metalloproteinase domain may affect the interaction between TIMP and MMP21 and ultimately affect MMP21 activity. Although MMP21-L277F was damaged by PROVEAN, PolyPhen2, and MutationTaster, this variant rescued the phenotypes caused by mmp21 knockdown in zebrafish (Fig. 4). This may be due to the leucine to phenylalanine substitution not affecting the acid-base status and having little effect on spatial configuration. Both glycine to glutamic acid substitution in p.G244 and lysine to glutamic acid substitution in p.K487 changed the charge of the amino acid residues which may have affected protein folding and function. The variant p.K487E is located in the hemopexin-like domain, which consists of four β-helices. The Hpx domain may confer substrate specificity and is involved in the recognition and catalytic degradation of ECM proteins [43]. Therefore, p.K487E may influence the degradation of MMP21 substrates. In addition, a pathogenic de novo dominant variant might drive the dominant-negative behavior by impairing the product of the wild-type allele. Our data suggest that the MMP21 variants p.G244E and p.K487E may act as dominant negatives, disturbing the cyclization process during zebrafish heart development, leading to the heart looping disorder phenotype. Also, MMP21-G244E and MMP21-K487E mutants are susceptible to abnormal left–right patterns and abnormal cardiac development, supporting the causal effect of MMP21 variants in the dextrocardia phenotype of HTX patients. Further studies are required to confirm whether MMP21-G244E and MMP21-K487E manifest a qualitatively similar but milder dominant-negative effect on MMP21 protein.

The genetic architecture of the HTX is complex. The current literature regarding the molecular basis of HTX implicates numerous genes in autosomal dominant, recessive, or X-linked modes of inheritance [44]. In addition, single-gene variants, aneuploidy, and pathogenic copy number variants have also been identified as causative factors of HTX [45]. In some instances, both autosomal dominant and recessive modes of inheritance have been reported for a single gene. For example, the NODAL antagonist DAND5 has been confirmed to pattern left/right body asymmetry by inhibiting the NODAL signaling pathway [46]. Specifically screening for the presence of DAND5 variants alone, a non-synonymous heterozygous variant of DAND5 (c.455G > A) was identified in two unrelated Portuguese patients with laterality defects and complex congenital heart defects, which first confirmed the autosomal dominant manner of DAND5 variant [47]. Interestingly, a homozygous disease-causing mutation in DAND5 (c.396_397dupCT, p.Y133Sfs*11) was screened out from a proband with HTX of Arab–Muslim descent, which as a novel recessive monogenic cause for HTX in humans [48]. These studies demonstrate that single-gene variants can serve as the molecular basis for the L-R pattern of HTX and congenital heart defects through both autosomal dominant and autosomal recessive modes of inheritance. Previous studies reported homozygous or compound heterozygous MMP21 mutants in patients with HTX [18, 19, 29–32], which indicates that biallelically damaging MMP21 variants are important in the left–right patterning of HTX. MMP21 variants currently identified in HTX patients with complex CHDs are summarized in Additional File 4. Of note, we detected only three heterozygous MMP21 mutants in this study. Zebrafish rescue experiments confirmed that simple heterozygous MMP21-G244E and MMP21-K487E are pathogenic to HTX. However, HTX is genetically heterogeneous, and previous studies have identified multiple genetic mutations involved in establishing left–right asymmetry, including NODAL signaling or primary ciliary dyskinesia-associated disease-causing genes. These destructive mutations could contribute to the pathogenesis of the cardiac and extracardiac phenotypes of patients with MMP21-mutated patients. Thus, we analyzed the exome sequencing data of MMP21-mutated patients to determine whether they had other potential HTX-causing genetic variants (Additional file 2: Table S2). In the patient with the MMP21-G244E mutation, two potential additional HTX-causing missense variants were found in ROCK2 (NM_004850, c.345G > C, p.Q115H, rs564194657) and NUP188 (NM_015354, c.5018C > T, p.P1673L, rs568477773). ROCK2B knockdown and ROCK signaling inhibition in animal models resulted in abnormal asymmetric gene expression and caused laterality defects in Xenopus, zebrafish, and chicken embryos [49–51]. In addition, previous studies have identified rare genic copy number variations and heterozygous variants of *ROCK2* in patients with HTX. NUP188 Morpholino knockdown in Xenopus strongly disrupted morphological left–right development, and a rare NUP188 duplication was identified in an individual with HTX and CHD [51, 52]. In HTX patients, no pathogenic missense mutations have been identified. Although the ROCK2 and NUP188 variants are not proven to be pathogenic, online pathogenicity analysis showed that these two variants may be destructive to protein function. So even though we confirmed that MMP21-G244E mutants failed to rescue heart looping defects in zebrafish embryos, we cannot exclude that the identified ROCK2 and NUP188 variants may jointly lead to the occurrence of cardiac and extracardiac malformations in this case. We haven’t found any other known HTX disease-causing gene variants in the MMP21 p.L277F mutated case. Given that the missense variant of MMP21 p.L277F successfully rescued the abnormal cardiac tube phenotype, the exact cause of this case is unknown. Interestingly, the patient with the MMP21-K487E variant had no genetic variations in other causative genes related to HTX, indicating a vital role for the simple heterozygous MMP21-K487E variant in the development of left–right axis patterning and heart looping.

Currently, few studies have focused on the molecular mechanisms of extracellular remodeling-related genes in establishing laterality during embryonic development in vertebrates. As a highly orchestrated process, embryonic development is inseparable from timed expression and activation of protease/antiprotease systems [53, 54]. MMPs are crucial in regulating vasculogenesis and epithelial-to-mesenchymal transition during organogenesis [55]. MMP21 is a zinc-dependent ECM remodeling endopeptidase involved in ECM degradation and embryonic development regulation [26, 27]. MMP21 is produced in various ectodermal structures in a spatially and temporally regulated manner during human and mouse embryonic development [56]. MMP21 could be detected in mice embryos aged 10.5, 12.5, 13.5, and 16.5 days after fertilization, with an expression peak at 13.5 days [56]. As for zebrafish embryos, WISH results showed that MMP21 expression was restricted to Kupffer’s vesicles when left–right asymmetry was established [29]. However, the substrates and interactors of MMP21 that control the LR pattern have not yet been clarified, especially during embryonic development, and identifying them will help uncover the mechanisms underlying the relationship between MMP21 loss-of-function and HTX pathogenesis as well as deepen our understanding of left–right asymmetry during embryonic development.

## Conclusion

In summary, our findings show that two de novo, heterozygous non-synonymous *MMP21* variants p.G244E and p.K487E affect the establishment of left–right asymmetry in zebrafish as well as play an indispensable role in heart looping, supporting the causal effect of heterozygous *MMP21* variants in the dextrocardia phenotype found in patients with HTX and CHDs. Our data provide new insight into the pathogenesis of HTX and further support the hypothesis that pathogenic heterozygous *MMP21* mutations contribute to the genetic underpinnings of HTX in humans. Moreover, our findings expand the mutation spectrum of *MMP21* in HTX and confer susceptibility to HTX with CHDs.

## Supplementary Information

**Additional file 1: Table S1.** Primers used for Sanger sequencing validation of *MMP21* variants, mutagenesis of mutant *MMP21* vectors, RT-qPCR, and construction of in situ hybridization probes.

**Additional file 2: Table S2.** Clinical information and HTX-related genetic variants in HTX patients with *MMP21* variants.

**Additional file 3: Table S3.** The variants of potential causative genes related to HTX identified in the clinical cohort.

**Additional file 4: Table S4.** The* MMP21* variants currently identified in HTX patients with complex CHDs.

## Data Availability

The dataset analyzed in the current study is available from the corresponding author according to a reasonable request.

## Abbreviations

HTX: Heterotaxy syndrome
CHDs: Congenital heart diseases
MMP: Matrix metallopeptidase
WT: Wild-type
SA: Single atrium
SV: Single ventricle
DORV: Double-outlet right ventricle
L-TGA: Levo-transposition of great arteries
CAVC: Complete atrioventricular canal
TAPVC: Total anomalous pulmonary venous connection
CSS: Cardiosplenic syndrome
RAI: Right atrial isomerism
PLSVC: Persistent left superior vena cava
PS: Pulmonary stenosis
VSD: Ventricular septal defect
PAD: Pulmonary artery dysplasia
AVSD: Atrioventricular septal defect
ECM: Extracellular matrix
TIMPs: Tissue inhibitor of metalloproteinases
AAT: Alpha-1-antitrypsin
