# Supplementary material for: De novo disruptive heterozygous MMP21 variants are potential predisposing genetic risk factors in Chinese Han heterotaxy children
Source: Hum Genomics. 2022 Sep 19;16:41. doi: 10.1186/s40246-022-00409-9 (PMC9484203; doi:10.1186/s40246-022-00409-9)
Supplement: Supplementary file 1 — Additional file 1: Table S1. Primers used for Sanger sequencing validation of MMP21 variants, mutagenesis of mutant MMP21 vectors, RT-qPCR, and construction of in situ hybridization probes. [file 40246_2022_409_MOESM1_ESM.docx]

**Supplementary material**

**Table S1 Primers used for Sanger sequencing validation of *MMP21* variants, mutagenesis of mutant *MMP21* vectors, RT-qPCR and construction of In Situ Hybridization probes.**

| **Primer name** | **Forward primer** | **Reverse primer** |
| --- | --- | --- |
| c.731G>A | AGCGGTTCCTGTCCAGATAC | GTCCCCATAGCGTGTCCTAT |
| c.829C>T | AGCGGTTCCTGTCCAGATAC | GTCCCCATAGCGTGTCCTAT |
| c.1459A>G | GCCTTTGAGTTGGACTGGTC | TGGGTTTTAACTTACAGTGCCA |
| Mut1-c.731G>A | GGGCCTTCGATGAGAGCGGGCAGGA | TCCTGCCCGCTCTCATCGAAGGCCC |
| Mut2-c.829C>T | CACGGGCATCAGCTTTCTCAAGGTGG | CCACCTTGAGAAAGCTGATGCCCGTG |
| Mut3-c.1459A>G | AACTTCAGTAATCCTCTCTGGATAAGAATTAAGTACTCGATTTCTGT | ACAGAAATCGAGTACTTAATTCTTATCCAGAGAGGATTACTGAAGTT |
| MMP21-human | AGGGCCTTAAACCAAATGTCT | TGGACGTCACAAACATCAAACCA |
| 18sRNA-human | AAACGGCTACCACATCCAAG | CCTCCAATGGATCCTCGTTA |
| mmp21-zebrafish | ATCCAGAGGCTGTACGGAGT | GCGTCCTGTTATTGCGGTTC |
| Actin-zebrafish | GTGCTGTTTTCCCCTCCATTGTTG | GTGTCATCTTCTCTCTGTTGGCTTT |
| cmlc2-zebrafish | GCTCTGGGTGTCCATGTAGG | AGGGCCTTAAACCAAATGTCT |
